# Constraining Forest Certificate’s Market to Improve Cost-Effectiveness of Biodiversity Conservation in São Paulo State, Brazil

**DOI:** 10.1371/journal.pone.0164850

**Published:** 2016-10-25

**Authors:** Paula Bernasconi, Stefan Blumentrath, David N. Barton, Graciela M. Rusch, Ademar R. Romeiro

**Affiliations:** 1 Institute of Economics—University of Campinas (UNICAMP), Campinas, Brazil; 2 Instituto Centro de Vida (ICV), Cuiabá, Brazil; 3 Norwegian Institute for Nature Research (NINA), Oslo, Norway; 4 Fellow of the Brazilian National Council of Scientific Research—CNPq, Brasília, Brazil; University of Waikato, NEW ZEALAND

## Abstract

The recently launched Brazilian “forest certificates” market is expected to reduce environmental compliance costs for landowners through an offset mechanism, after a long history of conservation laws based in command-and-control and strict rules.

In this paper we assessed potential costs and evaluated the cost-effectiveness of the instrument when introducing to this market constraints that aim to address conservation objectives more specifically. Using the conservation planning software Marxan with Zones we simulated different scopes for the “forest certificates” market, and compared their cost-effectiveness with that of existing command-and-control (C&C), i.e. compliance to the Legal Reserve on own property, in the state of São Paulo. The simulations showed a clear potential of the constrained “forest certificates” market to improve conservation effectiveness and increase cost-effectiveness on allocation of Legal Reserves. Although the inclusion of an additional constraint of targeting the BIOTA Conservation Priority Areas doubled the cost (+95%) compared with a “free trade” scenario constrained only by biome, this option was still 50% less costly than the baseline scenario of compliance with Legal Reserve at the property.

## Introduction

Until recent, direct regulation has been the most important type of policy for biodiversity conservation in Brazil. The main command-and-control (C&C) instrument for forest conservation is the Forest Code (FC), which amongst others requires, in the case of the state of São Paulo, a Legal Reserve (LR) encompassing 20% (up to 80% in the Amazon) of the property area covered with natural vegetation. Compliance has been low, with less than 10% of the farms claiming to have Legal Reserves [1] due to the misconception from the agriculture sector of an incompatibility between agriculture development and conservation [2].

In order to reduce the economic impact on landowners some flexible instruments were introduced and one of the most promising is a mechanism that allows the landowners who do not comply with the 20% LR requirements to offset their deficit in another farm which has more natural vegetation than required. This instrument is called Environmental Reserve Quotas (Portuguese acronym, CRA), hereafter referred also as “forest certificates”. The FC specifies that the CRA is a certificate of ownership equivalent to an area of native vegetation and the certificate may be issued for any area of native vegetation exceeding the LR requirement [3]. The expected role of the forest certificates market is to reduce the costs with compliance to the Legal Reserve requirements on private properties and to remunerate landowners who have natural vegetation on their farm above the Legal Reserve requirement. Trading in forest certificates also can have the potential to reduce social inequalities by allowing revenue transfers to regions that have low agriculture suitability and larger forest cover as such schemes are deemed to increase social justice [4].

The CRA is an incentive-based instrument included in a recent amendment of the Forest Code (2012), and still lacks full regulation defining for instance, the scope of the market. However, the current regulations establish that the CRA is only valid as a means of complying with the minimum requirement on land currently below the requirement (due to past deforestation) and that the areas must have equivalent extension and be part of the same Biome. In the case of São Paulo, the government is tending to define in its regulation that all CRA should be traded within the State to ensure a minimum area of Legal Reserve and reforestation within the State. Recognizing the cases where there is lack of supply of Legal Reserve surplus area for compensation, the Law allows that deforested areas are used to issue forest certificates, but ties the acceptance of the compensation to the prior reforestation of the area.

In addition to maintaining a certain proportion of forest cover, the state of São Paulo faces other conservation challenges. The state is the most industrialized and populated of Brazil, with 40 million inhabitants [5], and, at the same time, it has a vast biodiversity with many endemic species [6] and the two major biomes found in the State, Atlantic Forest and Cerrado, are recognized as global biodiversity hotspots [7] (S1 Fig) due to the high pressure and threat of extinction [8]. Only 14% of original area of Atlantic Forest [9] and 10% of Cerrado are left, hence conservation priority criteria are urgently needed in the design of conservation instruments.

The potential of market-based instruments such as CRA to contribute to a policy mix for biodiversity conservation has been recently assessed by many studies [3, 4, 10, 11, 12]. In Brazil, CRA studies have focused on the national level [11, 13, 10, 14], the state level [15, 16, 17] and some have had a theoretical approach [18, 19, 20]. Especially, Soares-Filho and colleagues provide a comprehensive review on the CRA market, analysing different regulatory scenarios and the consequences on carbon balance [3]. The study finds that the Atlantic Forest of São Paulo is one of the areas with potential carbon efficiency gains with CRA.

These studies have showed that although the instrument is not a panacea for forest biodiversity conservation, CRA may lead to gains in cost-effectiveness under specific ecological and socioeconomic circumstances [19, 3]. For instance, CRA has the potential to take advantage of differences in the agricultural suitability of the land and in the resulting opportunity costs of biodiversity conservation. Hence, the total area of Legal Reserves can potentially be achieved at lower overall opportunity cost [2], but the magnitude of these gains will rely on the level of constraint of the market, i.e. the narrower the market, the lower the variation in opportunity costs. However, to our knowledge, no studies have explicitly incorporated spatially explicit biodiversity conservation priorities to explore outcomes in terms of conservation effectiveness, nor have the potential impacts on the cost-effectiveness of varying CRA market constraints been explored in the literature. Thereby, there are trading possibilities along with their legal and operational constraints that could harness economic and environmental benefits [12] that have not yet been assessed. Another relevant aspect for policy design that deserves more attention is the potential of CRA in terms of cost reduction and gains in conservation cost-effectiveness compared to a baseline situation of Forest Law compliance, i.e. reforestation on own property.

The aim of the study is to assess the cost-effectiveness potential of trading in Legal Reserve within the state of São Paulo to simulate Legal Reserve compliance in three different scenarios with different configuration of policies and market restrictions to define the allocation of the forest LR certificates. The first scenario is the ‘Baseline’ where we simulate the compliance with LR based only on the Forest Law regulation (C&C), without CRA, i.e. reforestation *in situ* of LR deficits. In Scenario 2, we simulate the compliance with LR considering the CRA market under the current regulation, i.e. trade within São Paolo State and within biome of forest certificates from surplus and reforestation areas. In Scenario 3, a variation of the CRA market that constrains the CRA from areas for reforestation within the conservation priority areas designated by the BIOTA program [21], as a measure to increase conservation effectiveness.

Specifically, we test at the state scale, the hypothesis that the larger the spatial market for forest certificates, the greater the opportunity cost differentials and the greater the economic arbitrage opportunities in a market, which would result in smaller compliance costs, as already assessed at national scale [3]. Our related hypothesis is that if costs and conservation values are heterogeneous and positively correlated, increasing market size increases cost-effectiveness. Hence, the allocation of forest certificates by the CRA market will result in more forest area reforested in areas of top priority for conservation, leading to higher conservation effectiveness compared to the baseline scenario of *in situ* reforestation. We also hypothesize that restricting the market by a minimum set of conservation criteria can improve conservation cost-effectiveness since the correspondence of opportunity cost differentials and the gain in biodiversity conservation is not perfect [22]. Thereby, restricting the CRA market within conservation priority areas can disproportionately increase conservation cost-effectiveness compared to a scenario of a market without such restrictions.

## Materials and Methods

We first mapped the distribution of LR deficit and surplus and then used these maps to identify the area of ‘new LR’, i.e. reforestation areas within the own property *(in situ* compliance, scenario 1), and areas allocated through forest certificates (scenarios 2 and 3). In scenarios 2 and 3, both existing forest patches (surplus areas) and reforestation were considered. In scenarios 2 and 3, all surplus areas were allocated first to new LR, following the assumption that the CRA market would trade the existing surplus areas first, since they are less costly (no associated reforestation costs). The remaining LR deficit (here after, net deficit) in scenarios 2 and 3 was allocated to new LR by using a conservation planning tool, Marxan with Zones [23, 24]. It allocated the equivalent to the net deficit area to areas for reforestation within the State and within biome (scenario 2), and additionally, within high priority areas for conservation in scenario 3, while minimizing opportunity costs. After the allocation of new LR, we calculated the total opportunity cost of new LR and of *in situ* reforestation areas, and related costs to a metrics of conservation effectiveness in order to compare the cost-effectiveness among the different scenarios.

### Spatial distribution of deficit and surplus areas

We used a database from a state agricultural census (LUPA) [20] with data about the area with forest in each of the approximately 320 thousand Units of Agricultural Production (UPA), which is largely equivalent to a farm, for São Paulo State. We calculated the deficit and surplus of LR for each UPA, according to the reference value of 20% of LR required by law for the Atlantic Forest and Cerrado biomes. The database has a complete description of all areas of natural vegetation and fragile ecosystems on each property (e.g. forest, foodplain, swamps, etc.). Most of these should be protected as Permanent Protected Area (Portuguese acronym, APP) as required by the Forest Code. Also, the Forest Code in its Art.15 allows the addition of all APP in the calculation of Legal Reserve in the property, so we considered all those areas as LR.

In order to comply with confidentiality requirements, the UPAs were aggregated using a grid of hexagons of 500 hectare each, resulting in 50,600 planning units which were used as the spatial unit of the further analyses.

Landowners with properties sized below 4 fiscal modules, an unit of measurement used in Brazil that in Sao Paulo represents 20ha on average, which have less than 20% of LR do not need to buy ‘forest certificates’ nor to carry out reforestation in order to be compliant. However, not more than 80% of their property can be deforested. In addition, all the forest area in these properties can be considered a “surplus” for the CRA market. Thereby, these farmers can participate in the CRA market, but only as sellers. However, we were not able to eliminate the UPAs with less than 4 fiscal modules from the sample in the aggregated data we used.

### Calculation of opportunity cost

We use land value as a proxy for the opportunity cost of forest conservation, under the assumption that it reflects the present value of foregone agriculture production. We suggest that this is a practical proxy indicator for the willingness to accept payment for the CRA. A recent contingent valuation survey [3] showed that land prices are a proxy for the willingness to accept and to pay for a CRA, reflecting direct and indirect uses and non uses values for contracts of 30 years or more; the same study argued that for short-term contracts farmers tend to refer more often to land rent prices.

Land value data were based on summary statistics on market price per hectare from the bare land value (BLV) database [25], compiled semi-annually. In this database, for groups of municipalities (EDR), maximum, minimum and average land values are reported for different categories of land use suitability.

In order to associate land value from EDRs to land productivity we overlaid a map on administrative units and a map of land suitability for agriculture [26] (Figure A in S1 File), joining Land Categories with maximum, minimum and average land prices from the BLV land price database (Table A in S1 File). We assumed that at the municipality level, accessibility of the land was the main determinant of land prices within land-use classes. Thereby, the maximum, minimum and average land prices corresponding to each land categories class was assigned according to distance to infrastructure. Distance to infrastructure (here considered as roads and urban areas) was used as a von Thunen type proxy for accessibility to agricultural markets [27] (Figure B in S1 File). We applied a “cost distance” measure calculated with the r.cost module in GRASS GIS 6.4.2, that takes accessibility constraints of the landscape into account producing a friction map where rivers were treated as “barriers” and the friction of the terrain was defined as the squared slope in degrees.

A recent study of forest certificate market at the national level in Brazil used a similar procedure of assigning minimum and maximum land prices to three land-use categories [3]. In their study, municipalities with missing land prices were assigned land values through spatial (linear) interpolation to produce a continuous land value map. In our study, we replaced linear interpolation by a more heterogeneous and non-linear cost distance calculation. For each combination of municipalities and land use suitability classes, we then assigned the 25% of the pixels within this planning unit, which were closest to infrastructure (< 1^st^ quartile of the cost distance raster) to the respective max-value reported in the BLV database. The min-value was assigned to those 25% of the planning units with the greatest distance to infrastructure (> 3^rd^ quartile of the cost distance raster). The remaining intermediate cost distance locations were assigned average land market prices. The use of min-max land value statistics entails information loss relative to a time series of land market prices [3], but it is the only opportunity cost data available at a State scale. The lower and upper threshold of 25% of pixel cost-distance for assigning min and max land prices was chosen arbitrarily, as is the case of linear interpolation used in other studies. While opportunity costs as assigned here are expected to be both too high and too low at property level, the error distribution across a large number of analysis units is expected to be normally distributed (according to central limit theorem). However, assignment of thresholds still preserves the rank ordering of priority locations of Marxan with Zones.

**Fig 1:**
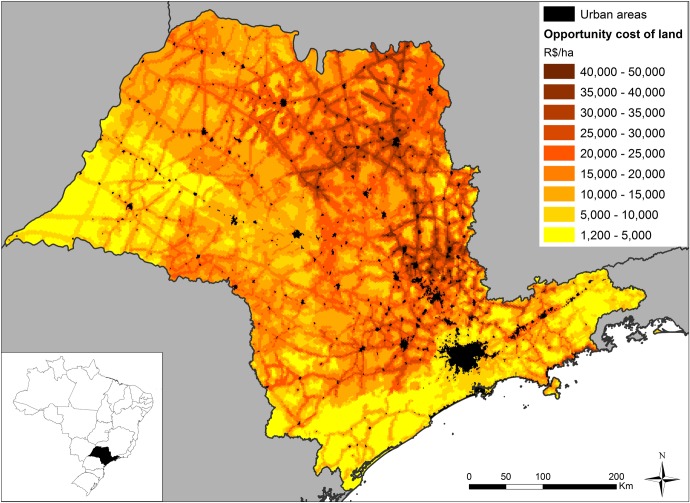
Distribution of opportunity costs of conservation in São Paulo. Opportunity costs of land varies from R$5,000 (lighter) to R$50,000 (darker) R$ per hectare in São Paulo, based on prices for cleared land. It was used as the cost criteria in the simulation.

The above steps resulted in a map (Fig 1) with costs per hectare varying from R$1,2 thousand to R$50 thousand. The resulting cost layer is based on potential agricultural returns and does not account for any forestry values that may be realised on properties.

### Criteria of conservation effectiveness

We used the map of Priority Areas for Conservation and Restoration produced by BIOTA [21] as a criteria for conservation effectiveness. This map, which has been the keystone of conservation planning in the State, compiles 20 years of biodiversity and ecological data. To date 15 governmental decrees and resolutions have based their recommendations for conservation priorities on the BIOTA/FAPESP program [6].

**Fig 2:**
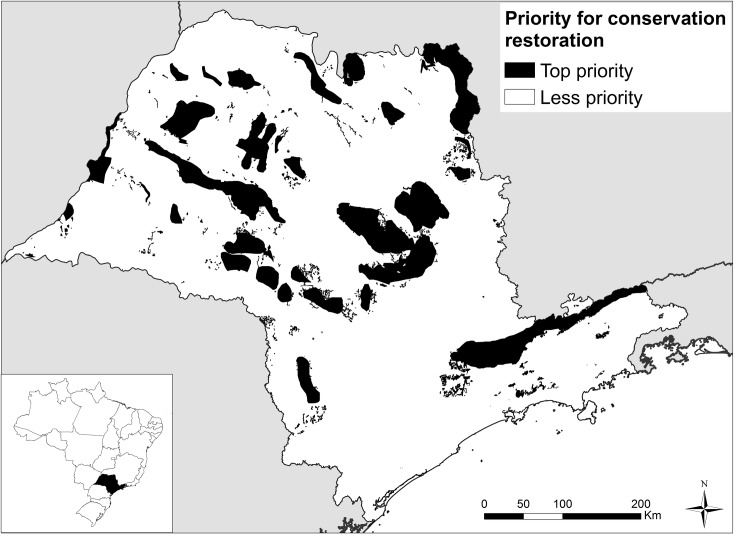
Priority areas for biodiversity conservation and restoration. Map adapted from BIOTA priority for conservation and restoration map [6], showing top priority areas for conservation and restoration in black (categories 5 to 8) and the rest of the state in white (categories 0 to 3). These two classes were used as criteria for conservation effectiveness.

The BIOTA map classifies the State of São Paulo in classes of priority for conservation and restoration ranging from 0 (low priority) to 8 (high priority). The amount of new Legal Reserves in the top priority classes, between 5 and 8 (Fig 2), were used as our indicator for conservation effectiveness, i.e. more forest certificates allocated on high priority areas entailed more effective conservation. In the case of net deficits that require reforestation, we argue that restoration performed on areas with higher priority for conservation would, *ceteris paribus*, also result in higher conservation effectiveness.

### CRA market allocation simulations

We used Marxan with Zones software [23, 24] to allocate land units to fulfil reforestation deficits at minimum cost of different market allocations of CRA in scenarios 2 and 3. We chose Marxan because the software:

1. finds solutions for allocating forest certificates at minimal costs, which represents the behaviour we expect of an efficient market with no transaction costs and where agents (landholders) have information regarding the distribution of opportunity costs and,
2. has the functionality to provide multiple near optimal solutions to meet conservation objectives [28]. This means that the algorithm does not produce one single optimal solution but many different alternatives an efficient market could allocate the required amount of Legal Reserve based on costs. We consider that this is a more realistic situation than to simulate a market as if there existed one optimal solution, which contrasts with an equilibrium model approach used in previous studies about CRA market [3]. Furthermore, the multi-solution output of Marxan with Zones provides additional information about whether there are many equally cheap or good alternatives (flexibility) and therewith how likely it is that the market will end up with a solution similar to those simulated, both in terms of costs and conservation outcomes.

In our scenarios, we used Marxan to allocate a fixed amount of a conservation feature that in our case, corresponded to a target set by the net deficit of LR area per biome. Hence, the application was used for the spatial allocation of the targeted feature within the available areas, constrained by the criteria of minimizing costs.

We used Marxan with Zones v2.1, for scenario allocation. The preparation and analysis of the spatial data were performed using ArcGis (ArcView v9.2), Quantum GIS v1.7.3 and GRASS v6.4.2.

### Scenario definition

In the baseline scenario we only considered reforestation of the deficit within each property as the option to comply with the Legal Reserve. In scenarios 2 and 3, we used a combination of forests certificates from surplus and reforestation areas.

Scenario 1: Baseline (*in situ* compliance): All area of deficits in the planning units were assumed to be reforested and set as new LR. The new LR area was then multiplied by the opportunity cost per hectare to get the total cost of the scenario. Next, we overlapped the new LR map with the BIOTA map to assess in which priority class for conservation the new LR were located.

Scenario 2: CRA market under current regulation: We considered that forest certificates would be allocated first to the surplus area (existing forests) within biomes. This area was multiplied by the corresponding opportunity costs to obtain the cost for new LR from surplus areas. Then, for each biome, we calculated the remaining deficit (net deficit) by deducting the surplus area from the deficit, in order to estimate the area needed for reforestation. Marxan with Zones was then used to allocate forest certificates for reforestation to fulfil the net deficit (the optimization target) in each Atlantic Forest and Cerrado, respectively. We defined two zones: the areas available and not available for allocation of forest certificates for reforestation. All planning units in the state of São Paulo (except urban areas, water bodies, existing forest remnants and existing protected areas) were considered to be available for the allocation to new LR using CRA from restoration areas. Each planning unit had two attributes: total opportunity cost (calculated as the opportunity cost per hectare multiplied by the area of each planning unit) and type of biome (Atlantic Forest or Cerrado). Marxan then selected planning units that reached the net deficit target in both biomes at least cost. We ran Marxan for 100 possible solutions of allocation of forest certificates with the lowest costs. For the calculation of the total opportunity cost for this scenario, we selected the best solution—i.e. the one achieving the net deficit target with the smallest opportunity cost—and added the calculated opportunity cost of the surplus LR area.

Scenario 3: CRA market restricted within BIOTA priority areas: We performed the same steps as in scenario 2, but, including the restriction of allocation within each biome in the state, and within areas of high priority for conservation/restoration (BIOTA classes 5 to 8). Thereby, the difference between scenario 2 and 3 is the constraint on where the forest certificates from restoration areas could be allocated. The planning units in the Marxan analysis had an extra feature indicating the priority class, and we excluded those planning units with class 0 to 4 from the units available for forest certificates allocation. The targets for Atlantic Forest and Cerrado were defined as the net deficit per biome after discounting the surplus areas, as in scenario 2.

The three scenarios were compared based on the level of opportunity costs, conservation effectiveness—the area (ha) of forest certificates allocated under high-priority conservation classes (5 to 8), and of cost-effectiveness, by dividing the area of high-priority classes by the total opportunity cost in each scenario. A graphic representation of the scenarios can be found at S2 Fig.

## Results

### Demand and supply

Our results show that natural vegetation covers 13.3% of all the rural area in State as defined by the LUPA [20] census, which represents a state-level deficit of 6.7% of Legal Reserve, equivalent to around 1.3 million hectares.

**Fig 3:**
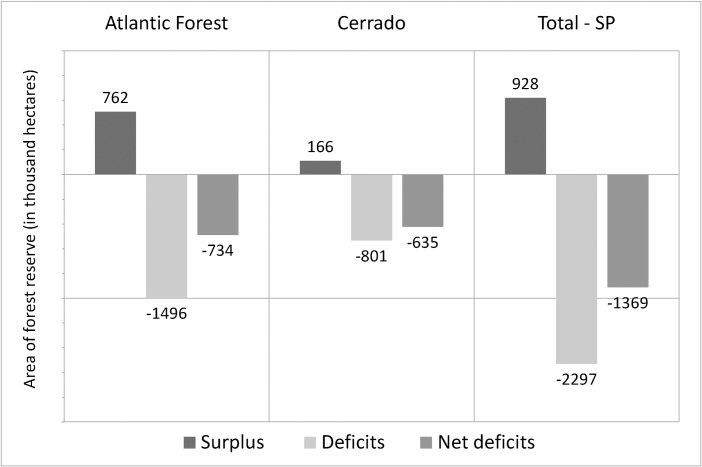
Area of deficit, surplus and net deficit per Biome in State of Sao Paulo. The bars show the area of surplus (dark grey). deficit (light grey), and net deficit (grey) of Legal Reserve (in thousand hectares) per Biome in State of Sao Paulo.

However, the distribution of the natural vegetation is very uneven within the State. Some areas are totally covered by natural vegetation, while others have 100% of crop plantation. The analysis at the planning unit level shows that 17,096 units have an area of natural vegetation larger than the required by law, a total of 928 thousand hectares of “surplus”, of which 762 thousand are at Atlantic Forest and 166 thousand at Cerrado (S3 Fig). On the other hand, 35,882 units have an area of natural vegetation smaller than required by law, with a total of 2.3 million hectares of “deficit” (Fig 3). This total deficit of Legal Reserve is distributed across 1.4 million hectares in the Atlantic Forest and 801 thousand hectares in the Cerrado (S4 Fig). The surplus/deficit ratio for the Atlantic Forest is 1/2 (one hectare of surplus for 2 hectares of deficit) and for the Cerrado is a ratio of 1/5 (one hectare of surplus for 5 hectares of deficit).

### Scenarios results

In all scenarios, the target of total deficit area (2.3 million hectares) was allocated finding planning units adding up to the same amount of area as “new Legal Reserves”. Scenario 1, without CRA, was the one with the highest market value, with total opportunity costs of R$37 billion. Scenario 2, that includes forest certificate trading constrained only by biome resulted in considerably lower costs, R$8.9 billion. The Scenario 3 including constraints for top priority areas for conservation, had a cost of R$17.4 billion (Table 1). These values do not represent the amount that the landowners will have to pay to be compliant (buying or renting CRA) as the value is only a proxy of the costs. The most important result is the relative value among scenarios.

**Table 1:** Summary of scenario results.

	New Legal Reserves				Effectiveness (hectares)	Cost-effectiveness (ha / million R$)
	Compliance using	Biome	Area (thousand ha)	Total Costs (million R$)		
**Scenario 1**	Reforestation	Atlantic Forest	1,496	21,3	-	-
		Cerrado	801	15,7		
	**Total**		**2,297**	**37,0**	**275,993**	**7,45**
**Scenario 2**	Surplus	Atlantic Forest	762	2,642	-	-
		Cerrado	166	1,121		
	Reforestation	Atlantic Forest	734	5,137		
		Cerrado	635			
	**Total**		**2,297**	**8,9**	**336,168**	**37,81**
**Scenario 3**	Surplus	Atlantic Forest	762	2,642	-	-
		Cerrado	166	1,121		
	Reforestation	Atlantic Forest	734	13,675		
		Cerrado	635			
	**Total**		**2,297**	**17,5**	**1,489,507**	**85,46**

The table shows the amount of new Legal Reserves (either by reforestation or surplus of forest from another property) in each biome and scenario. It also shows the total costs, effectiveness and cost-effectiveness ratio of each scenario.

**Fig 4:**
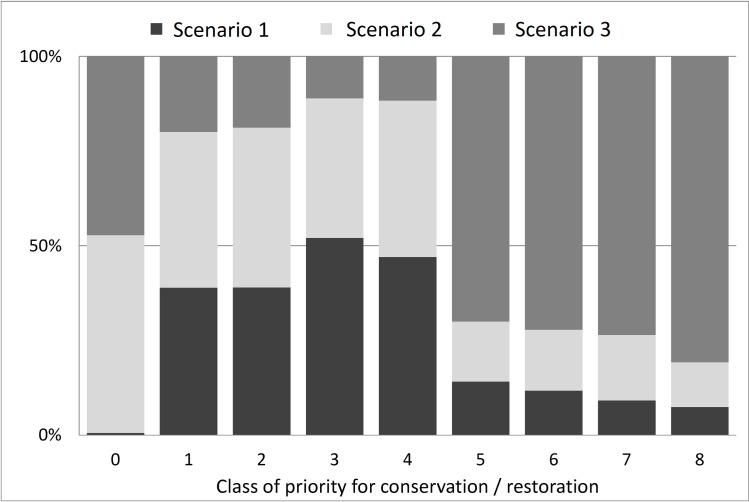
New Legal Reserves by classes of priority for restoration, by scenario. The bars show the relative distribution of new Legal Reserves selected in each class of priority for conservation (0 to 8, being 8 the highest), in each of the three scenarios.Scenario 2 had a similar result in terms of the area of new Legal Reserves in top priority classes (14%), but resulted in a higher representation of very low priority classes (i.e. increased representation of class 0 from 0.1% to 9%). In Scenario 3 more than 64% of the new Legal Reserves were located in classes of top priority for restoration, 5 to 8.

With regards to the representation of the new Legal Reserves in high priority areas for conservation, Scenario 1 had 38% of the new Legal Reserves concentrated in priority class 3, and 19% in priority classes 2 and 4. Only 12% of the new Legal Reserves were located in areas of top priority classes (5–8) (Fig 4 and Table B in S1 File).

**Fig 5:**
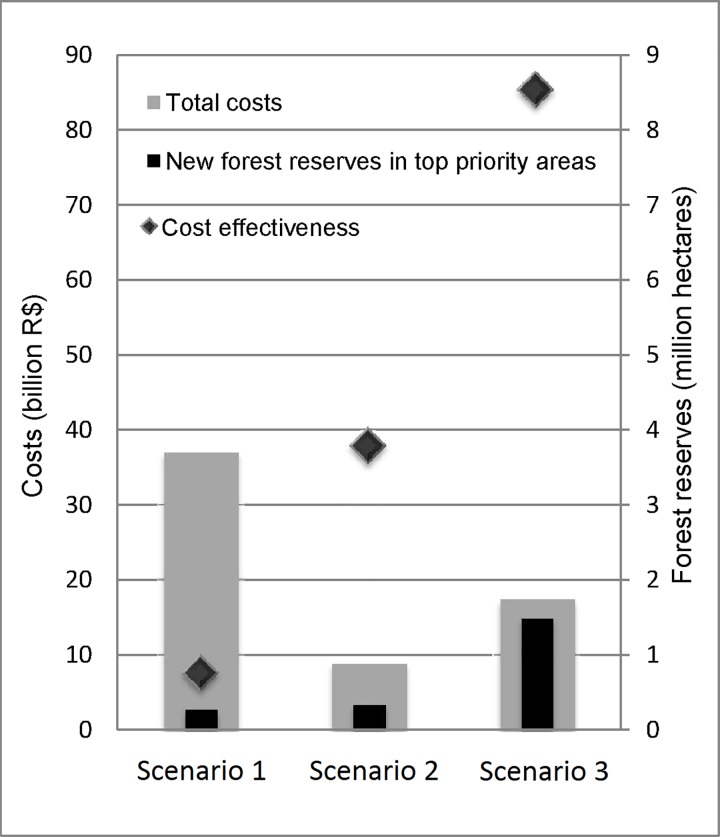
Total costs, conservation effectiveness and cost-effectiveness ratio, by scenario. The graph shows the total costs (grey columns, in billion R$) and the total area (black columns, in million hectares) of new Legal Reserves in top priority areas, by scenario. The rhombus show the ratio between these two values, resulting in the cost-effectiveness of each scenario (hectares of priority areas per million R$).

When compared to the baseline scenario, the Scenario 2 showed a reduction in costs of 76% and the Scenario 3, a reduction of 53% (Fig 5). Compared to the baseline scenario, Scenario 2 presents an increase in conservation effectiveness of 22%, while Scenario 3 presented a very high increase (440%), as expected by the inclusion of the constraint.

The Scenario 1 resulted in a cost-effectiveness of 7.45 high priority hectares/million R$ (Fig 5). The Scenario 2 resulted in a cost-effectiveness ratio of 37.81 high priority hectares/million R$ and the Scenario 3, 85.46 hectares/million R$.

The costs mentioned for Scenarios 2 and 3 include the best allocation of reforestation areas calculated by Marxan considering the lowest cost and the constraints imposed. However, we expect that a real market solution will likely be far from the best optimal solution. For this reason, we analysed the frequency of selection of each planning unit as new Legal Reserve for reforestation among the 100 possible Marxan solutions.

**Fig 6:**
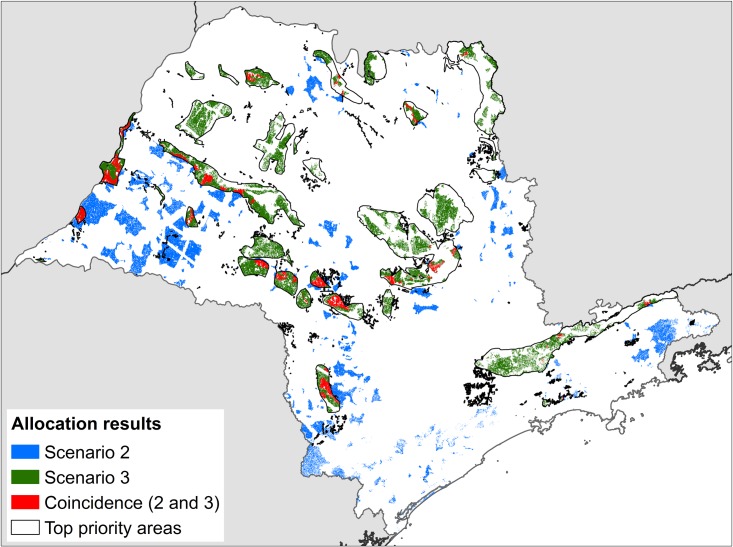
Allocation results of Scenarios 1 and 2. Results from Marxan show distribution of selected area for new Legal Reserves in the best run (lowest cost) of scenario 2 (blue) and scenario 3 (green). The map also highlights (in red) the coincidence areas selected in scenarios 2 and 3. The black polygons are the borders of the top priority areas for conservation / restoration.

In Scenario 2 (Fig 6), the areas selected in the lowest cost allocation of Marxan are concentrated in the western and central parts of the State, with some patches in the northern and eastern area. In Scenario 3, the areas selected in the best run are concentrated in the central and northern parts, with only 16% of coincidence areas between the two scenarios.

We summarized these results with a “flexibility index”, defined as the number of selected planning units divided by the average selection frequency (>0) of all planning units, in order to compare the availability of good alternatives. It resulted in a flexibility index of 805.3 for Scenario 2 and 872.7 for Scenario 3. This result indicates that despite the constraint of availability of areas in conservation priority areas, Scenario 3 has a larger set of available good alternatives compared to Scenario 2.

## Discussion

### High potential of improving cost-effectiveness

The results indicate high cost-effectiveness of the forest certificate trading and a high potential to both reduce compliance costs–as measured by opportunity costs—and improve the conservation effectiveness of the Legal Reserve compliance. The inclusion of forest certificate trading within biomes reduced compliance costs with Legal Reserve by 76% compared with a scenario of compliance with reforestation of deficit at the property without trade (Scenario 1). Although the inclusion of a new constraint targeting the BIOTA Conservation and Restoration Priority Areas almost doubled the cost (+95%) compared with a “free trade” scenario constrained only by biome, it was still 50% less costly than the baseline scenario of compliance.

Besides having the largest cost and the least efficient result in targeting conservation priority areas, the Scenario 1 has the disadvantage of leaving 762 and 166 thousand hectares of Atlantic Forest and Cerrado forest remnants surpluses, respectively, without protection by law as Legal Reserves. These areas are usually marginal agricultural lands, with very low opportunity cost and with conditions that have made them of limited interest to deforestation until now. But, many are still very important for biodiversity conservation as indicated by BIOTA. Also, compliance through reforestation could displace the demand of land for agriculture and some of the more productive of these forested areas could suffer an increased deforestation pressure due to leakage effects [29].

### Conservation outcomes of a market constraint

The proposed inclusion of a constraint on CRA market targeting the BIOTA priority areas simulated in Scenario 3 showed substantially larger conservation gains relative to the increase in costs, compared to the reference scenario with no forest certificates trading. This led to considerable increase in conservation effectiveness and resulted in the most cost-effective option. This result complement findings in a previous study [3] showing that while restrictions in market size raise total compliance costs, they also reduce the potential for carbon leakage, and are in agreement with studies indicating the potential for increasing conservation cost-effectiveness due to a disproportional correspondence between conservation gains and cost increments [22].

According to the selection frequency results, the selected areas for new Legal Reserves in Scenarios 2 and 3, overlap only in 16%. This result indicates that high priority areas for conservation are also more expensive (in opportunity costs), and that a constraint only within biomes will likely produce an outcome which does not fully reflect conservation priorities. This is not unexpected since areas of high conservation value are likely to coincide spatially with areas with high agricultural productivity, where the pressure of human activities is highest, and consequently, where the most threatened nature occurs. The result also illustrates the importance of a policy mix that combines market and regulatory instruments [30, 31], since market forces alone will tend towards nature protection areas only on lands that are unprofitable for agricultural production [4].

In the scenarios where trade is assumed, the option of buying forest certificates allocated in properties investing in reforestation above the required 20% of area protection is especially important in states such as São Paulo where the surplus area is smaller than the area of deficits, and where reforestation is necessary for compliance. However, it is still not regulated by law, and there are some questions under discussion at the State level: i.e. how much time after the plantation is needed for a reforestation area to be considered “reforested” and valid for trading in a forest certificate market? Lacking regulation on these issues makes opportunity costs estimations of reforestation options speculative at present as reforestation costs may vary from US$760 to US$20,000 per hectare [32] depending on the alternatives chosen. Also, considering the conservation importance, an area of forest remnants is often richer in biodiversity and have higher conservation value than a new planted forest. The researcher community also argues that even strict requirements about methods of plantation may be insufficient to guarantee the success of a reforestation project, i.e. to be self-sustaining in the mid- to long term [33]. Further studies should be conducted comparing the costs and benefits of forest certificate trading with the other options for compliance for landowners, such as natural regeneration and reforestation.

### Data limitation

The database used in the analysis imposed some limitations regarding the size of the properties and on the consequences of farm size for the legal reserve calculations. However, up to date it is the only-state wide and georeferenced database available with data for each property. Other studies have overcome limitations due to the lack of a unified land registry by using watersheds as a proxy for rural properties [3, 34]. Although this method has showed an acceptable level of uncertainty for national level analysis, we considered more accurate to use property data from LUPA [20] available for the state of São Paulo. The new national Environmental Rural Register (CAR), created by law in 2012, has the potential to be a great source of data for further analysis of economic instruments and environmental policy. Although it already represents 81% of the area subject to registration in Brazil [35] the database is still not publicly available.

### Implications for policy

Due to the current very low implementation of the Legal Reserve and pendency on regulation, trading in forest certificate must be considered more as a potential instrument than as an existing one. Besides its potential highlighted in our results, a number of caveats must be taken into account. First, the alternatives between a wide scope and little regulated market on one hand, and a spatially restricted and regulated trade scheme on the other, has to be considered carefully. As our study shows, the implementation of trading in forest certificates could reduce the costs for landowners without rendering cost-effective results for conservation. On the other hand, trading restricted within the same biome and watershed as the previous version of the Forest Code would reduce cost heterogeneity of land potentially participating and the possibilities of arbitrage, including the margins necessary to sustain intermediaries to make a trading scheme work. The issue of the scope of the market is relevant given that each of the Brazilian states must pass regulation with or without barring CRA trade beyond their borders, and even the State of São Paulo it is still under debate. The importance of the cost heterogeneity across properties for arbitrage on cost differentials between buyers and sellers depends on characteristics of landscapes in a market and equilibrium effects of the ratio of supply to demand. Using a market equilibrium model Soares-Filho and colleagues [3] demonstrate how total opportunity costs can actually decrease when trading is allowed with states in Amazonia with a large potential surplus of forest certificates. In their analysis, the large supply would seem to dominate the effects of cost-differential resulting from expanding the area of the market. Further analysis could document the optimal spatial extent of the market in terms of landscape heterogeneity and cost-differentials.

However, critical to the implementation of the Law is its conservation outcomes. Our analysis included a scenario showing one possible constraint inclusion that specifically targeted priority areas for conservation and very significantly increase cost-effectiveness. Based on biodiversity conservation values, our findings suggest more cost-effectiveness with spatially restricted markets, reinforcing the findings based on market equilibrium effects and carbon leakage [3]. There is the need for further study adding biodiversity constraints to the assessment of the size of forest certificate markets for Brazil as a whole, especially when analysed in a context of policy mixes with the potential to strengthen synergies between multiple objectives of conservation policies. Schröter and colleagues [22] for instance, studied spatial trade-offs among various ecosystem services provided by forests and showed that carbon sequestration targets could easily be achieved at low opportunity costs in contrast to certain biodiversity features, whose conservation was more costly, and argued for an inverse logic to the current international debate where carbon sequestration is targeted and (unmeasured) biodiversity conservation is seen as a co-benefit (see also [36]).

In addition, forest certificate trading requires institutional capacity to achieve the cost-effectiveness potential suggested by our modelling results. The new system to register the Environmental Rural Register, called SICAR, will be essential for administering Legal Reserve compliance and the forest certificates market. Third, perhaps the most important issue is the creation of demand. Biodiversity offset schemes such as this CRA market require a demand stimulated by a regulation of a cap or minimum reserve requirement [12, 18]. The environmental protection of such a system lies in the enforcement of the cap [37]. The forest certificate trading case in Brazil is an example of an existing economic conservation instrument that has not been implemented due to the lack of demand caused by lack of enforcement of the Legal Reserve. The last change in the Legal Reserve requirements, creating the CRA market has brought about the expectation of an increased enforcement of the law and has led to increased interest in compliance by property owners.

### Importance of a policy mix approach

These points highlight the importance of a policy mix approach for the design and implementation of cost-effective biodiversity conservation policies. In this approach, policymakers have a key role in combining different instruments to target the conservation objectives and to ensure its economic viability. Our modelling results show that CRA has potentially low increments in costs compared to the benefits for conservation, but it is potentially difficult to implement politically. One possible solution is to introduce targeted Payments for Ecosystem Services (PES) to increase conservation effectiveness of the market without increasing costs for landowners. For example, the State government could stimulate landowners to allocate the new Legal Reserves in priority areas offering to pay for the difference in opportunity costs that might exist. Alternatively, the State could buy land in priority regions to create new public Protected Areas and sell the quotas in the market, financing the creation of the protected areas and intervening in the allocation of the market. In some States where there is excess of supply of surplus LR the forest certificates could also be adapted to serve as a common financial mechanism for a wide variety of PES programs, in a concept of a X-CRA [3] multiplying environmental benefits of CRA beyond FC obligation. Several possibilities for instrument combinations should be addressed by policymakers and studied to find cost-effective and feasible solutions to fit each region.

## Conclusions

The results of our evaluation of the potential effect of the forest certificates market in the State of São Paulo show a clear potential to both reduce Legal Reserve compliance costs and improve conservation effectiveness compared to a pure command-and-control approach of Legal Reserves compliance without trading.

The inclusion of trading within each biome reduces compliance costs by 76% compared with the baseline of no trading. Although the inclusion of a new constraint targeting the Priority Areas almost doubled the cost (+95%) compared with Scenario 2 of “free trade” constrained only by biome, it was still 50% less costly than the baseline. The proposed scenario also showed substantially larger conservation gains relative to the increase in costs resulting in the most cost-effective option. Our analysis contributes to the literature on assessment of market-based instruments (MBI) to complement regulatory instruments reducing costs and/or improving effectiveness of the conservation policies using a policy mix analyses perspective. Our analysis of biodiversity conservation priorities and opportunity costs suggests that trading is more cost-effectiveness with markets restricted to targeting conservation priorities. Higher per hectare costs are offset by higher conservation effectiveness. Our findings complement those who show that more spatially restricted markets lead to higher prices, but also lower carbon leakage [3]. We also show how cost-effectiveness analysis of mixes of regulatory and market-based instruments can be assessed using Marxan with Zones, complementing other modelling approaches.

## Supporting Information

S1 Fig Landscape mosaic of State of São Paulo. The map shows the public protected areas, urban areas, forest remnants, water bodies, main roads and the original area of the two Biomes: Cerrado and Atlantic Forest. Source: SMA-SP, EMBRAPA, IF, ANA, IBGE, respectively.
(TIFF)

S2 Fig Graphic representing the scenario’s criteria and methods. (TIF)

S3 Fig Surplus of Legal Reserve distribution in São Paulo. Numbers are the amount of surplus in hectares on each planning unit classified by the sample quantiles (excluding zero surplus to better representation on a single class). The Atlantic Forest and Cerrado biomes are also represented at the map.
(TIF)

S4 Fig Deficit of Legal Reserve distribution in São Paulo. Numbers are the amount of surplus in hectares on each planning unit classified by the sample quantiles (excluding zero deficit to better representation on a single class). The Atlantic Forest and Cerrado biomes are also represented at the map.
(TIF)

S1 File File with supplementing figures and data. (DOCX)
