# Supplementary figures and images for: Constraining Forest Certificate’s Market to Improve Cost-Effectiveness of Biodiversity Conservation in São Paulo State, Brazil
Source: PLoS One. 2016 Oct 25;11(10):e0164850. doi: 10.1371/journal.pone.0164850 (PMC5079756; doi:10.1371/journal.pone.0164850)

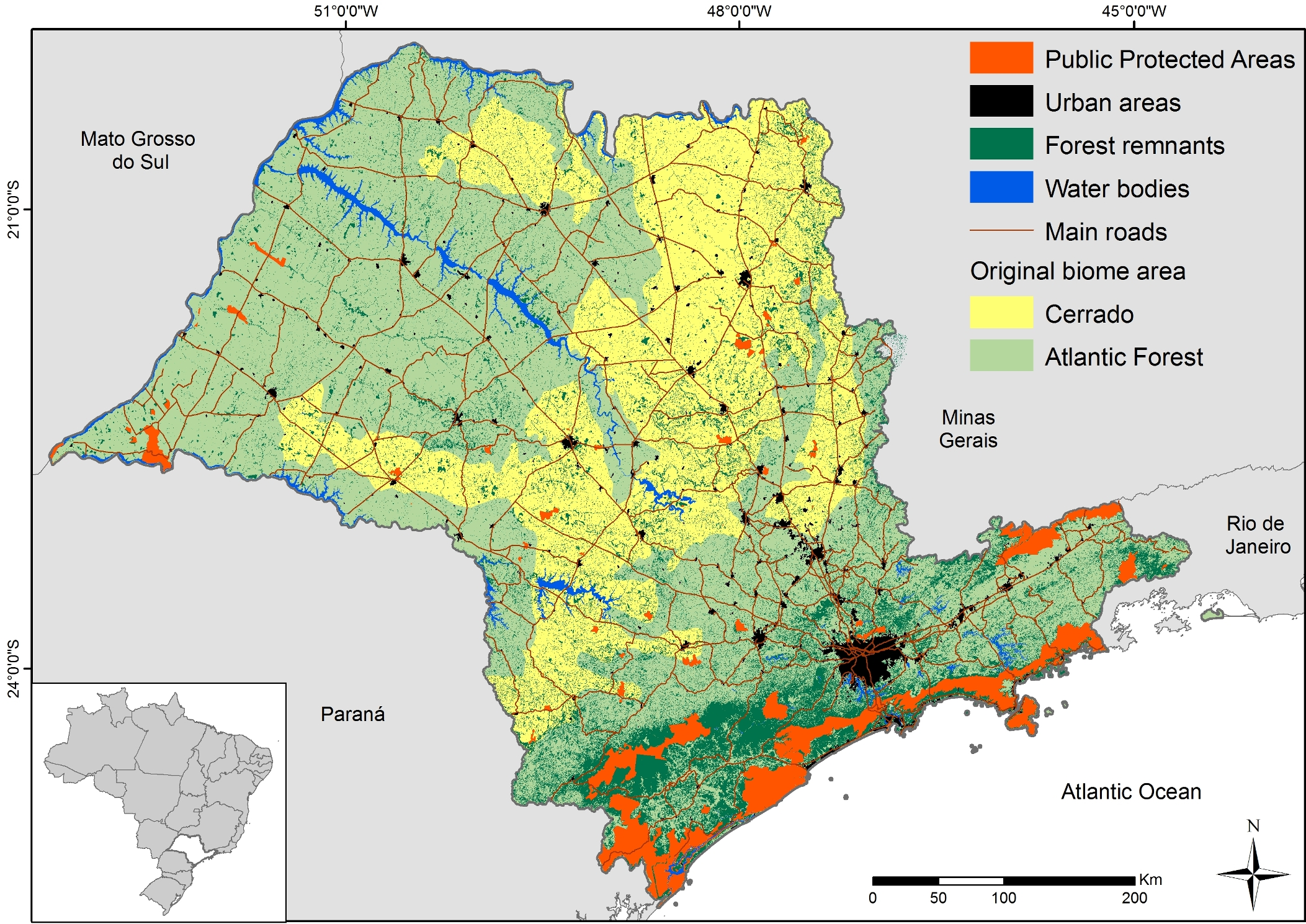

Supplement: S1 Fig — The map shows the public protected areas, urban areas, forest remnants, water bodies, main roads and the original area of the two Biomes: Cerrado and Atlantic Forest. Source: SMA-SP, EMBRAPA, IF, ANA, IBGE, respectively. (TIFF) [file pone.0164850.s001.tiff]

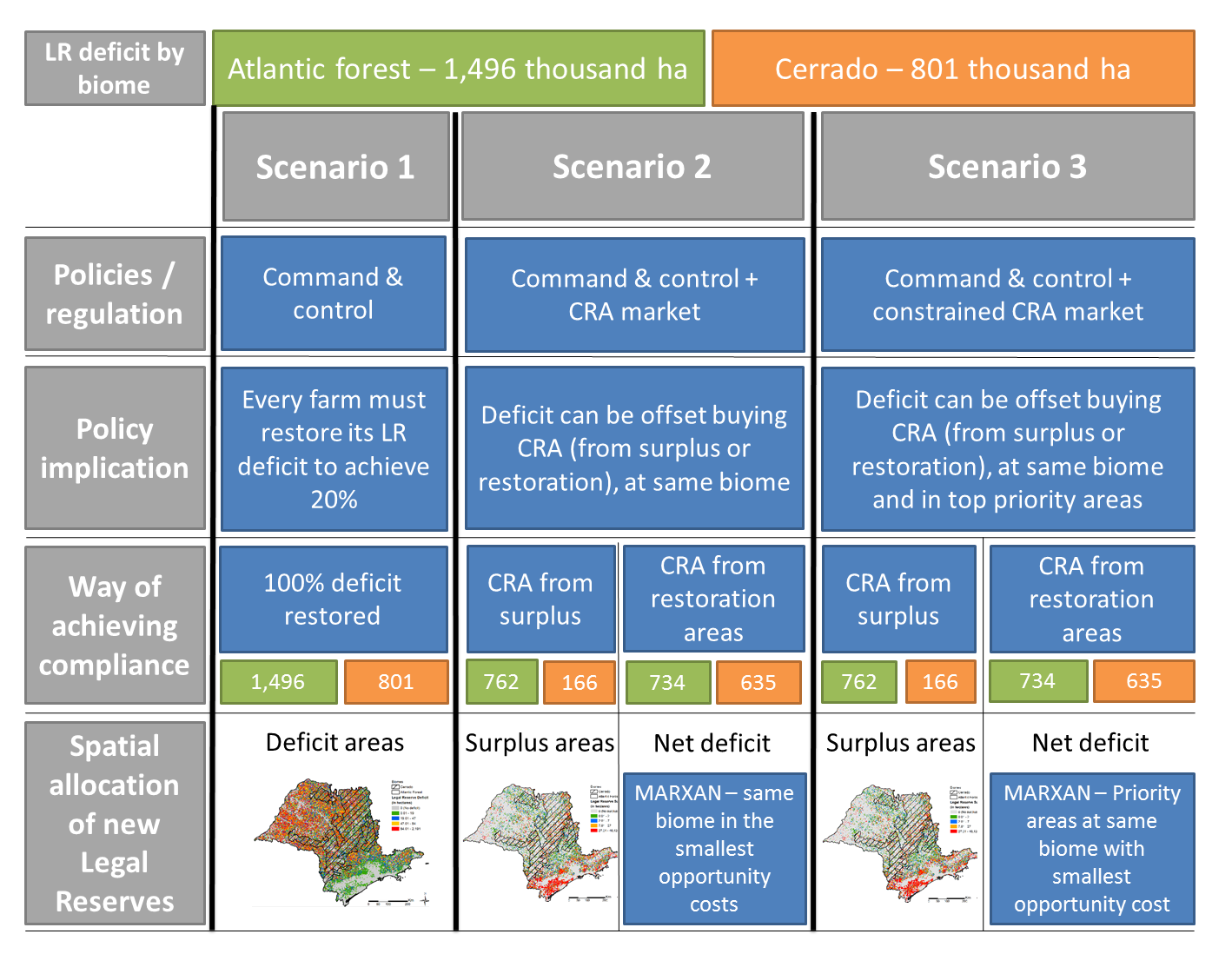

Supplement: S2 Fig — (TIF) [file pone.0164850.s002.tif]

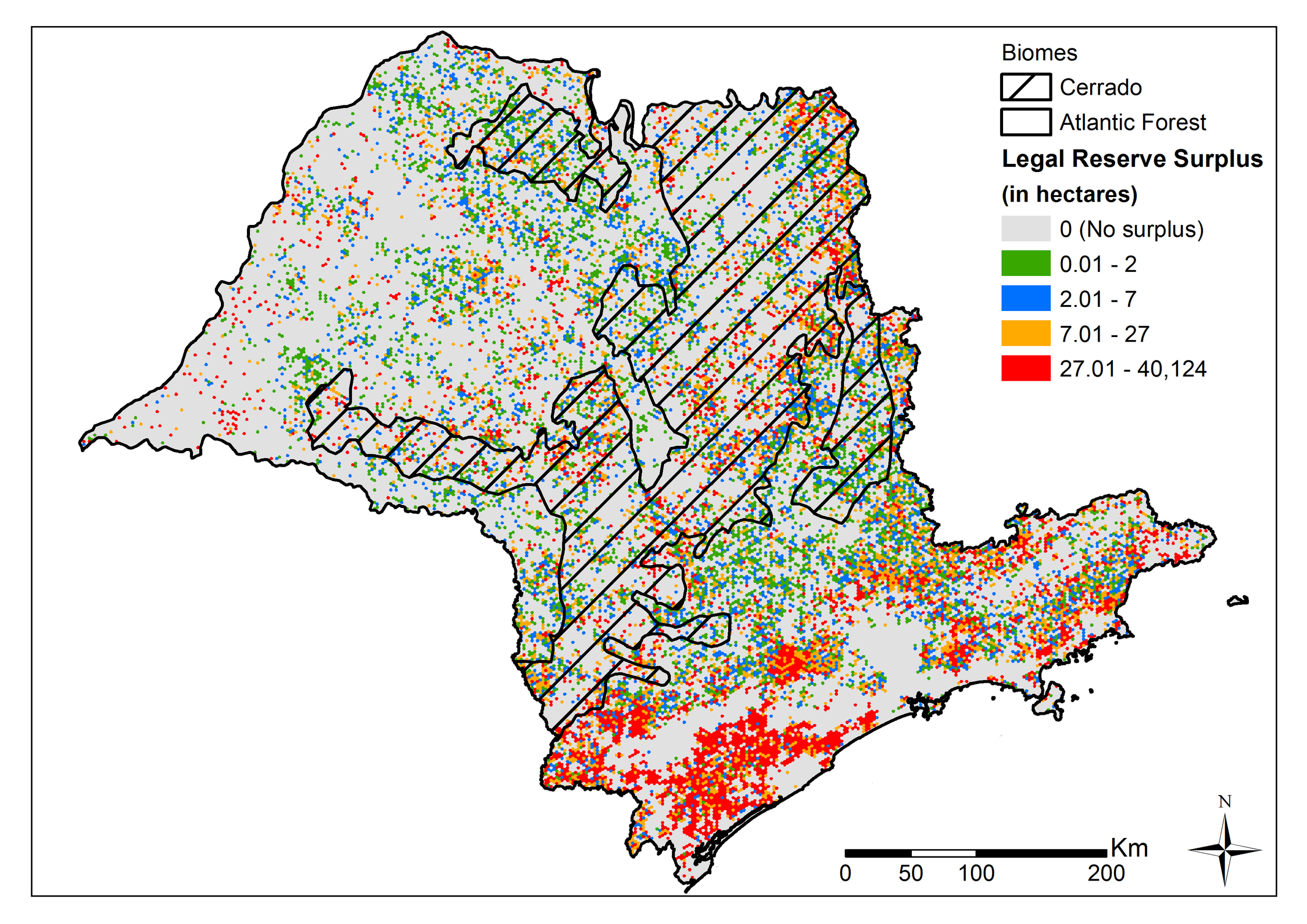

Supplement: S3 Fig — Numbers are the amount of surplus in hectares on each planning unit classified by the sample quantiles (excluding zero surplus to better representation on a single class). The Atlantic Forest and Cerrado biomes are also represented at the map. (TIF) [file pone.0164850.s003.tif]

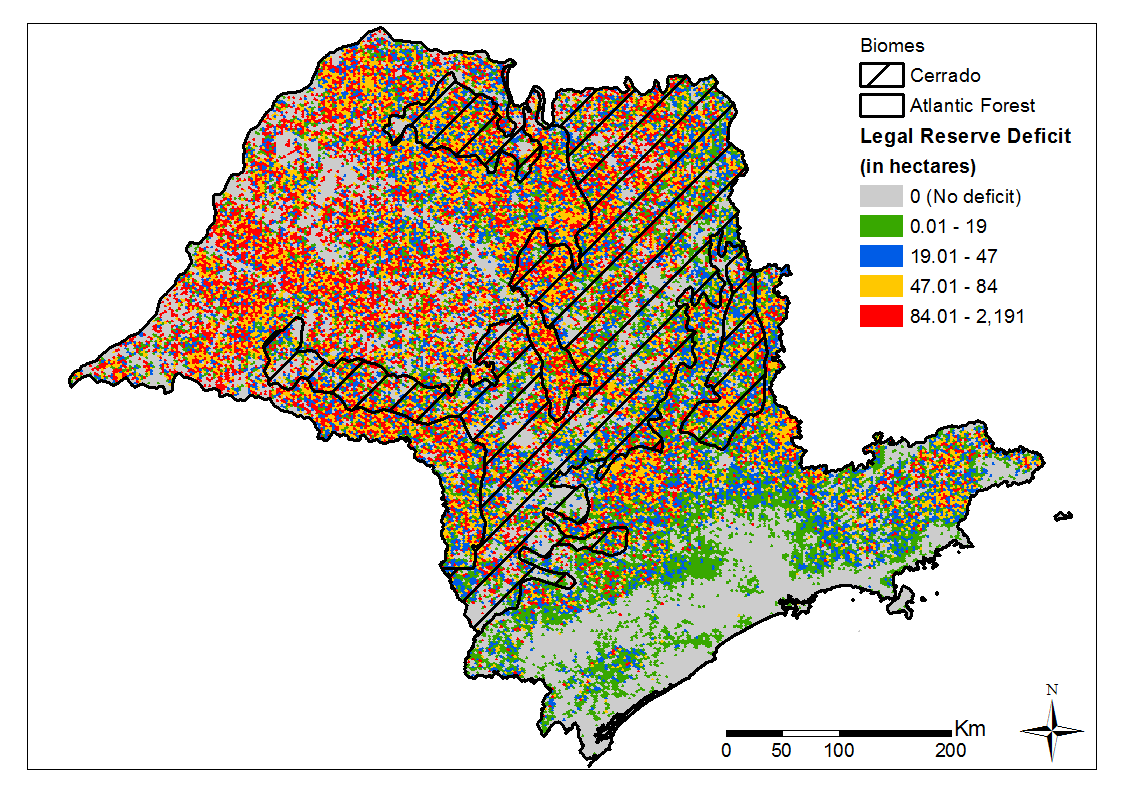

Supplement: S4 Fig — Numbers are the amount of surplus in hectares on each planning unit classified by the sample quantiles (excluding zero deficit to better representation on a single class). The Atlantic Forest and Cerrado biomes are also represented at the map. (TIF) [file pone.0164850.s004.tif]
