# Supplementary material for: Constraining Forest Certificate’s Market to Improve Cost-Effectiveness of Biodiversity Conservation in São Paulo State, Brazil
Source: PLoS One. 2016 Oct 25;11(10):e0164850. doi: 10.1371/journal.pone.0164850 (PMC5079756; doi:10.1371/journal.pone.0164850)
Supplement: S1 File — (DOCX) [file pone.0164850.s005.docx]

**S1 File. File with supplementing figures and data**

**DOI: 10.1371/journal.pone.0164850**

**Constraining forest certificate’s market to improve cost-effectiveness of biodiversity conservation in São Paulo State, Brazil**

**Fig A. Suitability for agriculture categories.** Digitalized and adapted from [26].

**Table A. Land categories (Bare Land Values) and Suitability for agriculture.**

| **Land categories (Bare Land Value – BLV) [25]** | **Suitability for agriculture [26]** |
| --- | --- |
| **First-rate Land for cultivation (*Terra de cultura de primeira*)**: potentially suitable for annual crops, perennials and other uses, which supports intensive management of cultural practices, soil preparation, etc. Presents average to high productivity, able to mechanize, flat or with slight slope, and the soil is deep and well drained. | **Group I:** Good suitability for agriculture cultivation. |
| **Second-rate Land for Cultivation (*Terra de cultura de segunda*):** although potentially suitable for annual crops, perennials and other uses, has more limitations than first-rate land. Can show mechanization difficulties due to the slope terrain. However, the soil is deep, well drained, with good fertility needing, sometimes some fertilizer. | **Group II:** Regular suitability for agriculture cultivation. |
|  | **Group III:** Limited suitability for agriculture cultivation. |
| **Land for pasture (*Terra para pastagem*):** unsuitable for crops, but potentially suitable for pasture and forestry. It is a land of low fertility, flat or hilly, with simple to moderate requirements for the conservation and management practices. | **Group IV:** Good to regular suitability for pasture. |
| **Land for reforestation (*Terra para reflorestamento*):** unsuitable for perennial crops and pastures, but potentially suitable for forestry and conservation, whose topography vary from flat to very hilly and may have very low fertility. | **Group V:** Good, regular, limited or no suitability for forestry and natural pastures. |
| **Land of field (*Terra de Campo*):** land with natural vegetation, primary or not, with limited possibilities of use for pasture or agriculture, whose best use is for fauna and flora conservation. | **Group VI:** No suitability for agriculture use, except in special cases. Suitable for preservation of fauna and flora or recreation. |

Correspondence between land categories from Bare Land Value database [25] with the categories of Suitability for agriculture map [26].

**Fig B. Data used for infrastructure (roads and urban areas) and to produce “friction” map.** Source IBGE**.**

**Table B. Amount of new Legal Reserves by class of priority for restoration, by scenario.**

| **Class of priority for conservation / restoration** | **Scenario 1** | **Scenario 2** | **Scenario 3** |
| --- | --- | --- | --- |
| 0 | 2,036 | 193,248 | 174,767 |
| 1 | 267,081 | 281,876 | 136,786 |
| 2 | 446,542 | 483,189 | 215,464 |
| 3 | 875,412 | 619,658 | 186,926 |
| 4 | 429,996 | 377,803 | 107,059 |
| **5** | **193,836** | **217,745** | **961,191** |
| **6** | **69,625** | **94,922** | **427,293** |
| **7** | **12,328** | **23,179** | **98,816** |
| **8** | **203** | **323** | **2,206** |

The table shows the amount in hectares of new Legal Reserves selected, by class of priority for restoration and by scenario. The bold classes are the top priority (5 to 8), considered for conservation effectiveness criteria.
